# Supplementary material for: Experts fail to reliably detect AI-generated histological data
Source: Sci Rep. 2024 Nov 19;14:28677. doi: 10.1038/s41598-024-73913-8 (PMC11577117; doi:10.1038/s41598-024-73913-8)
Supplement: Supplementary file 1 — Supplementary Information 1. [file 41598_2024_73913_MOESM1_ESM.pdf]

## Supplementary Figures

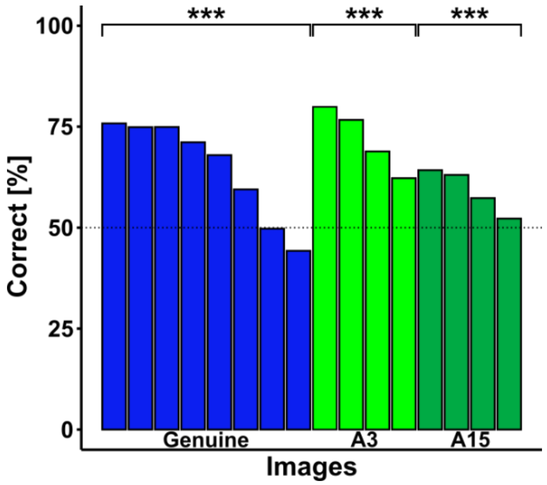


**Supplementary Fig. 1. Proportion of correct responses for individual images by image category.** All three categories consist of images with significantly differing proportions of correct classifications. Chi-Square within category: $p_{genuine} < 0.001$, $p_{A3} < 0.001$, $p_{A15} < 0.001$. Data from naïve and expert participants were pooled.

a)

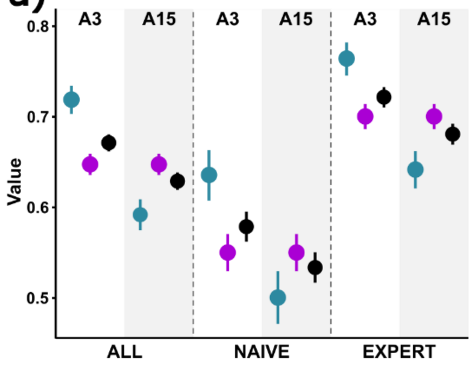


b)

| | | Sensitivity | | | Specificity | | | Accuracy | | |
|---|---|---|---|---|---|---|---|---|---|---|
| | | Point | Lower | Upper | Point | Lower | Upper | Point | Lower | Upper |
| ALL | A3 | 0.72 | 0.7 | 0.73 | 0.65 | 0.64 | 0.66 | 0.67 | 0.66 | 0.68 |
| | A15 | 0.59 | 0.57 | 0.61 | 0.65 | 0.64 | 0.66 | 0.63 | 0.62 | 0.64 |
| NAIVE | A3 | 0.64 | 0.61 | 0.66 | 0.55 | 0.53 | 0.57 | 0.58 | 0.56 | 0.6 |
| | A15 | 0.5 | 0.47 | 0.53 | 0.55 | 0.53 | 0.57 | 0.53 | 0.52 | 0.55 |
| EXPERT | A3 | 0.76 | 0.75 | 0.78 | 0.7 | 0.69 | 0.71 | 0.72 | 0.71 | 0.73 |
| | A15 | 0.64 | 0.62 | 0.66 | 0.7 | 0.69 | 0.71 | 0.68 | 0.67 | 0.69 |

**Supplementary Fig. 2. Performance metrics by experience and amount of training data. a)** Sensitivity (turquois), Specificity (violet), and accuracy (black) calculated separately for artificial images synthesized based on 3 or 15 training images. Data are displayed as point estimate and confidence intervals. **b)** Table displaying numerical values of statistics shown in a, providing point estimates and confidence intervals. Rounded to two decimals places. Note that specificity depends on true negatives (correctly classified genuine images) and false positives (incorrectly classified genuine images) but not on artificial images, resulting in identical specificity values for A3 and A15 images within each group.

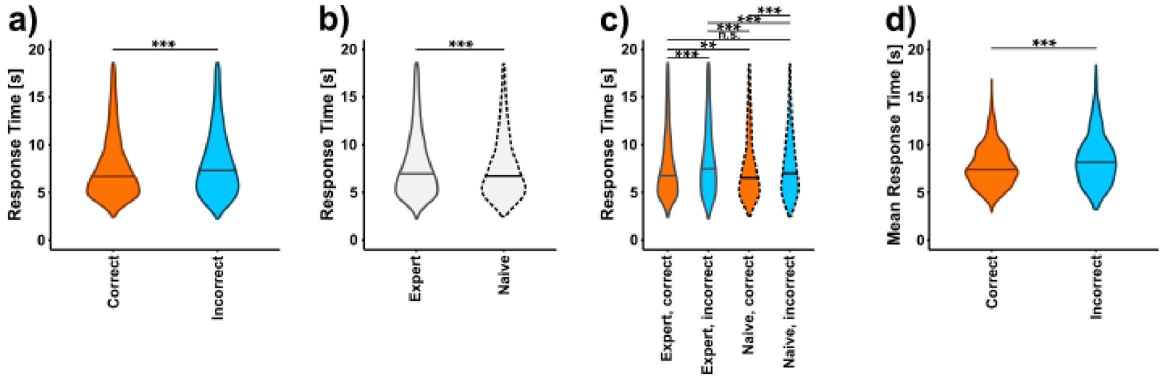


**Supplementary Fig 3. Response time effects are preserved after removing outliers.** Same data as in Figure 3 but after removing outliers, defined as response times beyond the last data point within 25th percentile - 1.5*IQR or 75th percentile + 1.5*IQR of overall response times, respectively (i.e. outliers are defined as data points outside boxplot whisker range of overall response times). This procedure resulted in a range of included response time values between 2.24 and 18.57 seconds. **a)** Correct responses take less time than incorrect responses (Wilcoxon rank sum test $p < 0.001$). **b)** Experts take longer to classify images (Wilcoxon rank sum test $p < 0.001$). **c)** Interaction between classification and experience (pairwise Wilcoxon rank sum test with Bonferroni correction: $p_{n,c\text{-}n,i} < 0.001$, $p_{n,c\text{-}e,c} = 0.0035$, $p_{n,c\text{-}e,i} < 0.001$, $p_{n,i\text{-}e,c} = 1$, $p_{n,i\text{-}e,i} < 0.001$, $p_{e,c\text{-}e,i} < 0.001$. **d)** Within-participant response times for correct and incorrect responses (Wilcoxon signed rank test $p < 0.001$). All panels display data as violin plots with the median indicated as horizontal line.
